# A closed conformation of the *Caenorhabditis elegans* separase–securin complex

**DOI:** 10.1098/rsob.160032

**Published:** 2016-04-13

**Authors:** Gudrun Bachmann, Mark W. Richards, Anja Winter, Fabienne Beuron, Edward Morris, Richard Bayliss

**Affiliations:** 1 Division of Structural Biology, The Institute of Cancer Research, London SW7 3RP, UK; 2 Department of Molecular and Cell Biology, University of Leicester, Leicester LE2 9HN, UK; 3 Astbury Centre for Structural Molecular Biology, Faculty of Biological Sciences, University of Leeds, Leeds LS2 9JT, UK

**Keywords:** chromosome segregation, mitosis, protein purification, electron microscopy

## Abstract

The protease separase plays a key role in sister chromatid disjunction and centriole disengagement. To maintain genomic stability, separase activity is strictly regulated by binding of an inhibitory protein, securin. Despite its central role in cell division, the separase and securin complex is poorly understood at the structural level. This is partly owing to the difficulty of generating a sufficient quantity of homogeneous, stable protein. Here, we report the production of *Caenorhabditis elegans* separase–securin complex, and its characterization using biochemical methods and by negative staining electron microscopy. Single particle analysis generated a density map at a resolution of 21–24 Å that reveals a close, globular structure of complex connectivity harbouring two lobes. One lobe matches closely a homology model of the N-terminal HEAT repeat domain of separase, whereas the second lobe readily accommodates homology models of the separase C-terminal death and caspase-like domains. The globular structure of the *C. elegans* separase–securin complex contrasts with the more elongated structure previously described for the *Homo sapiens* complex, which could represent a different functional state of the complex, suggesting a mechanism for the regulation of separase activity through conformational change.

## 1. Introduction

The stages of the eukaryotic cell cycle are defined on the basis of chromosomal events and are referred to as G1, S, G2 and M phase. A cell in G1 phase commits to divide in the presence of favourable growth conditions, or growth signals, and enters S phase, the period when DNA synthesis takes place. During the synthesis process, connections between the newly replicated DNA molecules, called sister chromatids, are established [1–3], thus allowing the dividing cell to unambiguously identify chromatids as sisters. Once the chromosomes have been successfully duplicated the cell enters G2 phase. During mitosis, the dividing cell faces the crucial task of accurately segregating complete copies of its genome into a pair of daughter nuclei. The highly conserved cohesin complex holds the sister chromatid together and contains four core subunits: the kleisin family protein Scc1, two subunits of the structural maintenance of chromosomes Smc1 and Smc3, and the accessory subunit Scc3 [4]. Together, the core subunits form a ring-like structure that is thought to topologically encircle the DNA helices of the two sister chromatids [5–7]. A protease named separase dissolves the cohesion between the sister chromatids by cleaving Scc1 at the onset of anaphase [2,8–10].

Although separase is expressed throughout the cell cycle [11], it is inactive during most stages of the cell cycle owing to complex formation with its inhibitor securin [8,12,13]. This stable complex persists until shortly before the onset of anaphase when securin degradation is initiated by the anaphase-promoting complex/cyclosome (APC/C) [11,12,14]. While securin inhibits separase, it also plays a positive role in promoting its function. Experiments in budding yeast and human cells have demonstrated that securin is needed for full separase activity after itself has been degraded [13,15–17], indicating that securin is a chaperone for separase. This is supported by results from *Schizosaccharomyces pombe* and *Drosophila melanogaster* in which the absence of securin is lethal as it leads to an apparent lack of separase activity [14,18]. Several lines of evidence suggest that securin stabilizes separase: the accumulation of overexpressed separase has been reported to require co-expression of securin [19]; separase levels are over fourfold reduced in securin^−/−^ cells [17]; and the protein levels of budding yeast separase are three times lower in G1, when securin cannot be detected in the cell, than in other phases of the cell cycle [11]. The contribution of securin to separase stability is harder to ascertain in higher eukaryotes in which the levels of separase also fluctuate due to protein instability following autocleavage [19].

Separases are large proteins with molecular weights ranging from 140 to 240 kDa, with a few exceptions including *Drosophila* homologues. They belong to clan CD of cysteine peptidases, and are related to caspases and gingipain [20]. The catalytic activity of separases resides in their well-conserved C-terminal half, a region predicted to contain a domain common to caspases [21]. This domain harbours the strictly conserved histidine and cysteine residues needed for catalytic function [10,22]. In caspases and gingipain, the histidine and cysteine residues are brought into juxtaposition by association of the two hydrophobic beta sheets that bring the two amino acids close enough to one another to form the catalytic dyad [23,24]. In addition to the caspase-like domain, the C-terminal region is also predicted to contain a Death domain [21]. The N-terminal region of separase is thought to consist of Armadillo (ARM) or HEAT motifs that form α-helical repeats [25,26]. The C-terminal domain is separated from the N-terminal half by an unstructured central stretch (a ‘hinge region’). Pull-down studies reveal that the N- and C-terminal halves of both human and budding yeast separase form a complex [13,25]. Moreover, in yeast, the entire N-terminal region seems to be necessary for catalytic activity of the C-terminal caspase-like domain [13].

Securin proteins have extremely divergent primary sequences and, consequently, they can be challenging to identify through bioinformatics approaches [14,18,27,28]. Human securin is natively unfolded with only a small, transient helical region [29,30]. The region of securin that binds and inhibits separase has been identified in several systems, including fission yeast [31], *D. melanogaster* [25] and budding yeast [13].

A number of biochemical studies have been carried out to map the separase and securin interaction, which is stable even under high-salt conditions [32]. Interaction studies firmly establish that the C-terminal part of securin and the N-terminal region of separase are important for complex formation [11,13,25,26,30]. However, structural studies on the separase–securin complex have been limited by the difficulty of generating substantial quantities of stable sample. Indeed, the only published study is a low-resolution electron microscopic (EM) analysis of the human complex that showed a flexible, elongated structure [26].

We set out to study the separase/securin complex from *Caenorhabditis elegans*, in which the separase protein appears to be smaller and more highly ordered than homologues from other model organisms. Here we present the expression, purification and biochemical characterization of this complex*.* Negative stain EM and single particle reconstruction revealed the overall shape of the complex at a resolution of approximately 24 Å corresponding to a globular two-lobed structure that differs substantially from that of the equivalent complex from humans. The *C. elegans* structure can be interpreted in terms of homology models of the N-terminal HEAT repeat domain and C-terminal death and caspase-like domains of separase.

## 2. Results

### 2.1. Bioinformatic analysis and domain structure assignment of *Caenorhabditis elegans* separase

**Figure 1.**
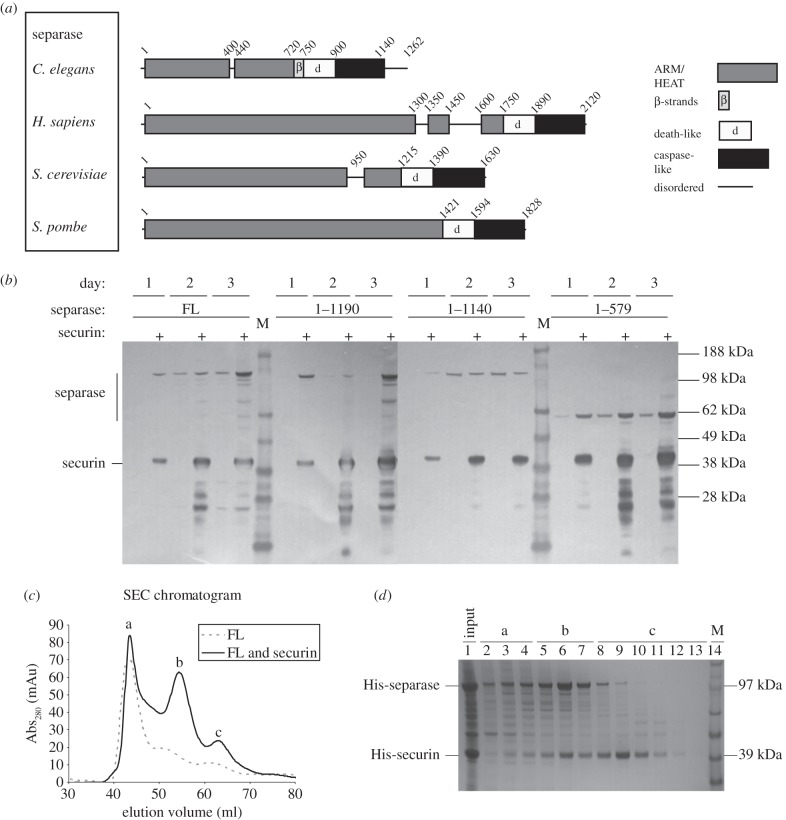
Securin increases the expression of full-length and N-terminal fragments of separase. (*a*) Predicted domain structure of separase proteins. (*b*) Expression of His_6_-separase and co-expression with His_6_-securin in Sf9 insect cells. Samples were taken on days 1–3 from a culture of Sf9 cells infected with virus containing His_6_-separase constructs only, or viruses containing both His_6_-separase and His_6_-securin constructs. Samples were analysed by Western blot, using α-His_6_ primary antibody, and by loading twice the amount of separase only samples versus separase and securin samples. Both separase and securin are indicated. Lane M indicates the molecular weight marker. (*c*) Size exclusion chromatogram of full-length (FL) *C. elegans* separase when expressed and purified with securin. Three peaks are visible which correspond to void (peak a), separase–securin complex (peak b) and securin alone (peak c). Purification of separase alone leads to an accumulation of the protein in the void. (*d*) Fractions from SEC of separase co-expressed with securin were analysed by SDS–PAGE. Lane 1 is the input sample. Peak a consists of separase protein (lanes 2–4). Peak b consists mostly of separase–securin complex (lanes 5–7). Un-complexed securin elutes as peak c (lanes 8–12). Lane 14 shows a molecular weight marker (M).

Secondary structure prediction was carried out on separase from *C. elegans, H. sapiens* and *S. cerevisiae* using PsiPred [33]. The N-terminal region was predicted to be mostly α-helical with varying helix lengths. Previous published analysis suggests that this region of human separase is composed of ARM or HEAT repeats [25,26]. Fold recognition predictions carried out using HHpred [34] and Phyre^2^ [35] matched the N-terminal regions of separase from *H. sapiens*, *S. cerevisiae* and *C. elegans* to helical and super-helical structures such as Tpr repeats, and, with less confidence, ARM or HEAT repeats. These three types of repeat all give rise to right-handed solenoid structures: whereas ARM regions have three helices per repeat, HEAT and Tpr regions have two helices per repeat and are distinguished at a sequence level or by the usually larger curvature of HEAT regions [36]. However, in the case of *C. elegans* separase, sequences did not clearly fit a consensus and were only partly modelled by either programme, indicating that the N-terminal part of separases contains a non-canonical fold of ARM/HEAT repeats or a super-helical structure that is not part of either fold. This is likely to be due to these repeats often being highly diverged, making the prediction of the positions of HEAT or ARM domains challenging [37]. The N-terminal region of *C. elegans* separase also contains a predicted disordered region from residue 400 to 440, as well as three beta-strands from residue 720 to 750 (figure 1*a*). The analysis revealed a feature seemingly unique to the worm homologue: whereas the C-terminus of most homologues lies at the end of the caspase-like domain, the *C. elegans* homologue has an additional 120 residues C-terminal to the caspase-like domain. The core region of the *C. elegans* separase homologue, comprising the α-helical repeat region and the caspase-like region, is smaller than that of other separase proteins, and so we investigated its suitability as a model system for structural studies.

### 2.2. Co-expression of separase and securin stabilizes the complex

Attempts to express separase, or parts of *C. elegans* separase, in *Escherichia coli* did not yield any soluble protein that was folded and stable. However, it has been suggested that securin functions not only as an inhibitor of separase but also as its chaperone [17,19], and co-expressing the inhibitor with separase was therefore attempted. However, co-expression of N-terminal separase constructs with full-length securin as well as co-expression of N- and C-terminal separase constructs in *E. coli* did not give rise to any change in expression pattern or stability of the proteins.

We expressed different constructs of *C. elegans* separase in Sf9 insect cells, corresponding to full-length and three C-terminal truncations. In each case, expression yields were increased by co-expression with securin as analysed by SDS–PAGE, and we loaded twice the amount of separase-only samples per lane versus separase and securin samples to aid visual analysis (figure 1*b*). The increase in expression yields for the *C. elegans* homologues ranged from approximately two times more (construct 1–1140) to around eight times more (construct 1–579) separase expressed, confirming reported observations that securin is required for accumulation of the protease [17,19].

We carried out large-scale purifications of separase alone and in complex with securin, and used size exclusion chromatography (SEC) to evaluate the suitability of the samples for structural studies. SEC of full-length separase alone showed a large peak corresponding to the void volume, indicating the presence of soluble aggregates of separase (figure 1*c*, dotted line, peak a). A proportion of the separase/securin complex also eluted in the void volume, but two further peaks in the chromatogram became more apparent (figure 1*c*, solid line, peaks b and c). Peaks b and c correspond to separase/securin complex and securin, respectively (figure 1*d*).

### 2.3. *Caenorhabditis elegans* securin is a disordered protein that interacts with separase through its C-terminal region

**Figure 2.**
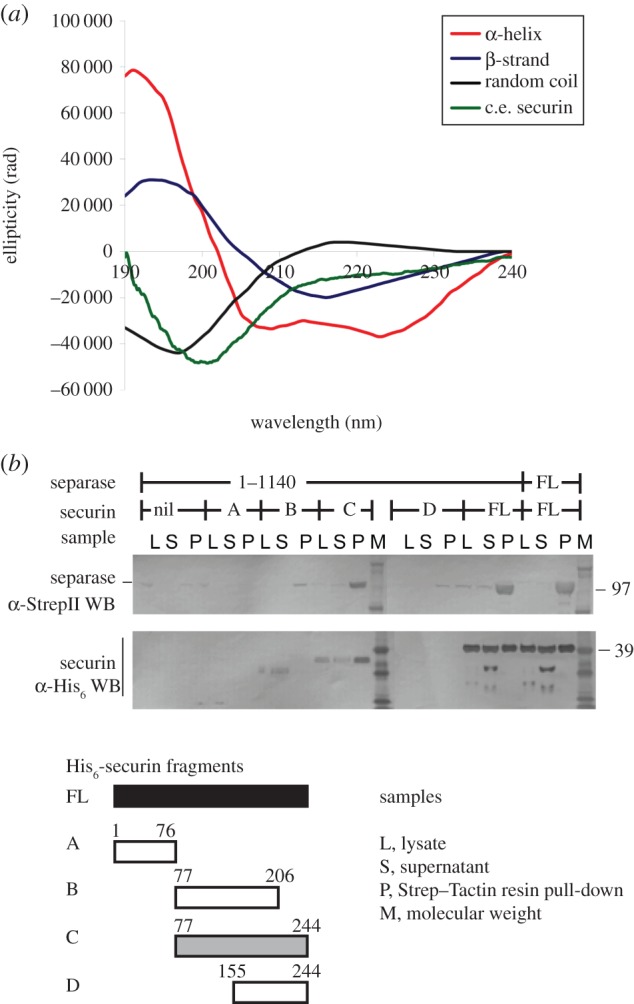
Structural and biochemical analysis of *C. elegans* securin. (*a*) *E. coli-*expressed *C. elegans* securin was analysed with CD spectroscopy. Spectra of secondary structure CD standard curves are shown for comparison. (*b*) Separase accumulation is highest when co-expressed with full-length (FL) securin and 77–244. Separase and securin fragments, as well as the full-length proteins, were co-expressed in Sf9 cells. After 72 h of expression the cells were harvested, lysed (samples ‘L’) and the insoluble fraction separated. The soluble fraction (samples ‘S’) was incubated with Strep–Tactin resin to bind the StrepII-tagged separase (pull-down, samples ‘P’). Samples were analysed with SDS–PAGE, and proteins visualized with Western blots to detect separase (top blot) and securin (bottom blot). Truncation mutants of securin, and FL protein are shown schematically at the bottom of the panel, and shaded by their propensity to form a robust complex with separase (black, most robust; white, no detectable complex).

Previously, it was shown that human securin is an intrinsically disordered protein [29,30]. We expressed and purified His_6_-tagged securin from *C. elegans*, and analysed the purified protein using circular dichroism (CD) spectroscopy to experimentally determine the degree of secondary structure in the protein. A CD spectrum of the protein was recorded from 190 to 260 nm at 25°C (figure 2*a*) and subsequently analysed for quantitative estimation of the secondary structure content. The results revealed that *C. elegans* securin contains 6% *α*-helices, 6–8% β-strands, 4–8% turns and 76–84% random coils. The minimal number of secondary structure elements in *C. elegans* securin, along with the published results that *H. sapiens* securin is also an unfolded protein [29], suggest that disorder is a conserved feature of securin proteins, at least when overexpressed in the absence of separase.

Bioinformatic analysis unveiled a very uneven charge distribution in the *C. elegans* securin sequence: its N-terminus (amino acid 1–76) contains predominantly positively charged amino acids (Lys, Arg), whereas the remainder of the protein (amino acid 77–244) contains a large number of negatively charged amino acids. The C-terminal region (207–244) has a cluster of predicted α-helices. To define a minimal separase-binding fragment of securin, batch purification experiments on StrepTactin resin were carried out using truncated securin proteins co-expressed with separase (aa 1–1140). Four different fragments of securin were expressed (1–76, 77–206, 77–244 and 155–244), and their expression and interactions were evaluated using SDS–PAGE and Western blots (figure 2*b*; electronic supplementary material, figure S1*a*). Only the full-length securin and 77–244 fragment formed stable complexes with separase. Securin fragments 1–76 and 77–206 did not interact with separase, whereas expression of the 155–244 fragment could not be detected.

### 2.4. Purification of the full-length separase–securin complex

**Figure 3.**
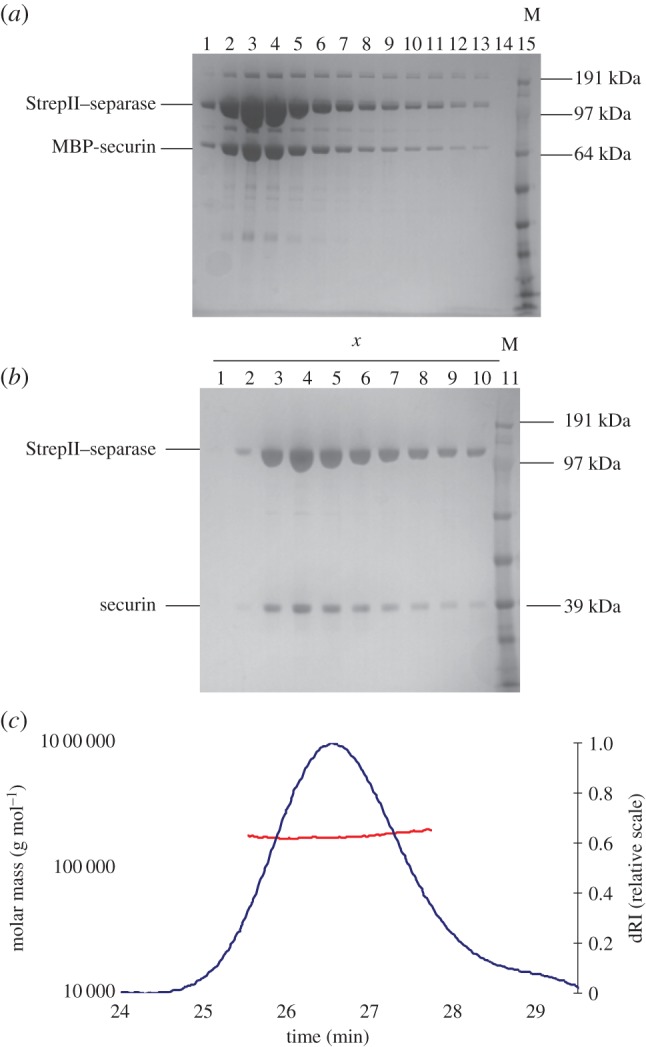
Purification of the separase–securin complex. (*a*) First purification step: elution fractions from a Strep–Tactin resin analysed by SDS–PAGE. (*b*) SDS–PAGE analysis of fractions corresponding to the main peak (*x*) of the SEC shown in electronic supplementary material, figure S1*d*. (*c*) SEC–MALS analysis of the separase/securin complex. Normalized dRI signal (blue line, right *y*-axis) was plotted against elution time and shows one peak confirming a monodisperse solution. The molar mass distribution (red line, left *y*-axis) confirms that the elution peak consists of one species with the molecular weight of 174 kDa.

Our original purification strategy used His_6_-tagged separase, but we found that imidazole strongly destabilized the thermal stability of the separase–securin complex (electronic supplementary material, figure S1*b*), and so we focused on optimization of protocols for purification of complex to homogeneity based on strep II-tagged separase (figure 3*a*; electronic supplementary material, figure S1*c*). The size exclusion elution profile of the separase–securin complex shows one peak, which contains both separase and securin and indicates complex formation (electronic supplementary material, figure S1*d*; figure 3*b*). Multi-angle light scattering (MALS) in conjunction with SEC was used for determining the size distribution and the accurate molecular mass of the complex [38]. SEC–MALS measurements confirmed the complex as a single species and gave an estimated molecular mass of 174 kDa (figure 3*c*), consistent with a 1 : 1 complex of separase (molecular weight of 144.2 kDa) and securin (molecular weight of 27 kDa).

### 2.5. Three-dimensional structure of the separase/securin complex

**Figure 4.**
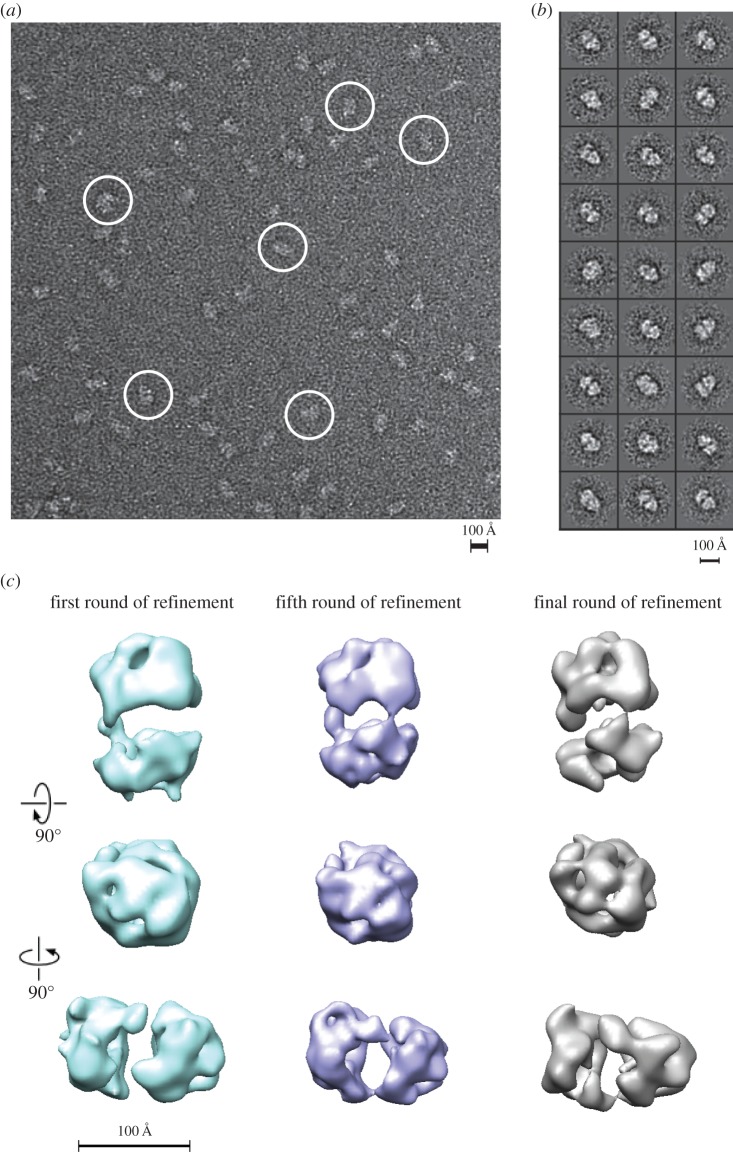
Electron microscopic analysis of negatively stained separase–securin complex. (*a*) A characteristic micrograph of freshly purified full-length *C. elegans* separase–securin complex is shown. The complex was bound to glow-discharged, carbon-coated quantifoil grids and stained for EM using 2% w/v uranyl acetate. Examples of molecular views are circled. (*b*) Reference-free two-dimensional class averages of the separase–securin complex represent characteristic molecular views of the complex with a high signal-to-noise ratio. Reference-free class averages of the *C. elegans* separase–securin complex were generated by refine2d from EMAN. (*c*) Refinement progress of the separase–securin model from angular reconstitution. The model is shown in three orthogonal views after the first (sea-green) and fifth (blue) round of refinement, as well as the refined model (grey) after a total of 10 refinement rounds. The models were contoured to give a mass of 174 kDa.

**Figure 5.**
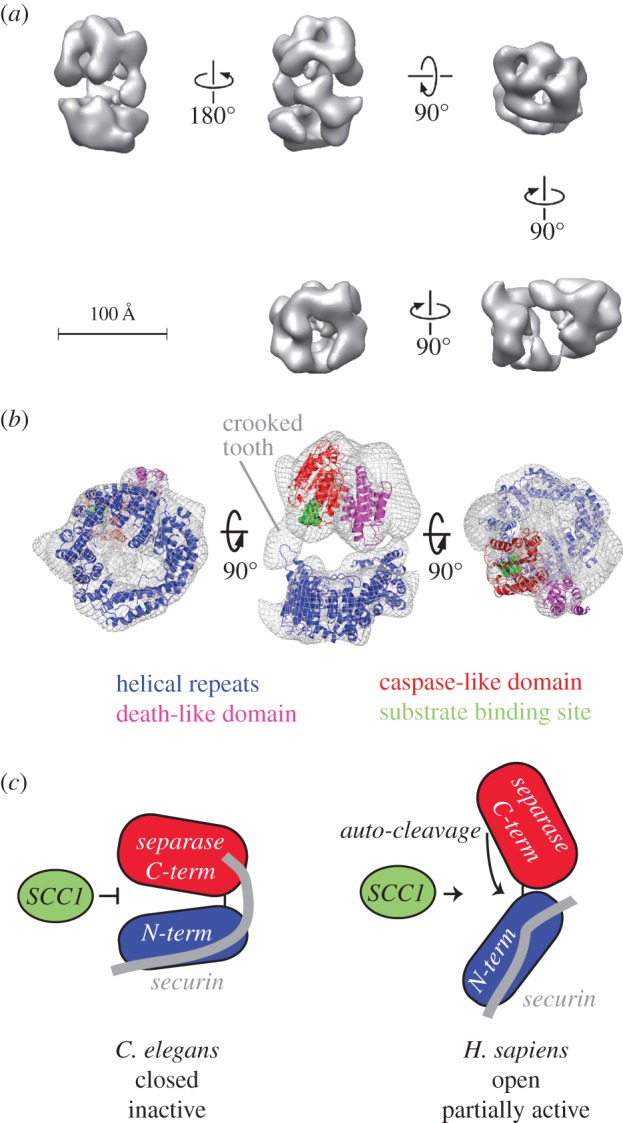
Structural model of the *C. elegans* separase–securin complex. (*a*) The refined *C. elegans* separase–securin structure shown in different views. This model shows intricate connectivity and a good amount of detail. The two structural domains are clearly distinguishable, as well as the central cavity. The model was contoured to give a mass of 174 kDa. (*b*) Mesh representation of the separase–securin complex and modelled separase domains, colour-coded; helical repeats (blue), death-like domain (pink), caspase-like domain (red) and Scc1 peptide substrate (green). (*c*) Schematic models of the separase/securin complex from *C. elegans* (this study) and *H. sapiens.* A closed, inactive form of the complex is consistent with its biochemical properties, such as inability to bind Scc1 protein substrate. We hypothesize that the open form of the human complex represents a partially active state of the complex, in which autocleavage occurs and Scc1 binding might be possible.

Transmission electron microscopy in conjunction with single particle analysis was carried out to gain structural insights into complex formation between separase and securin. Negative stained samples of full-length *C. elegans* separase–securin complex gave rise to molecular images (figure 4*a*, encircled) of consistent dimensions and with sufficient detail for three-dimensional analysis. Reference-free class averages typically appear divided into two lobes, differing in size and shape, separated by a central region of stain accumulation (figure 4*b*). An initial three-dimensional map of the *C. elegans* separase–securin complex was generated in C1 symmetry (i.e. no internal symmetry) from class averages with projection angles assigned by angular reconstitution in Imagic. The structure was then subjected to 10 cycles of refinement, consisting of multireference alignment, three-dimensional reconstruction and reprojection (figure 4*c*; electronic supplementary material, figure S2). The final, refined three-dimensional map displayed with a threshold consistent with a mass of 174 kDa, as determined by SEC–MALS (figure 3*c*), has a complex, globular shape with sufficient detail to show two structural lobes and their connectivity (figure 5*a*). The resolution of the refined map was calculated as approximately 24 Å (electronic supplementary material, figure S3). A notable feature of the map is a central cavity with dimensions of approximately 60 Å × 40 Å that separates the two structural lobes. The lobes are connected by a thin linker.

We next set out to determine a plausible arrangement of separase and securin in the complex. The larger lobe in the top half of the map (figure 5*b*, middle image) represents approximately 54% of the total mass of the complex, or 93.5 kDa, whereas the smaller lobe has an estimated molecular mass of 80.5 kDa. We generated a model of the helical repeat region of separase using i-Tasser [39]. This model has a ‘lock-washer’ shape made up of HEAT repeats that matches quite well the smaller lobe of the map (electronic supplementary material, figure S4*a*). The agreement between this model and the map density was further improved using the molecular dynamics flexible fitting (MDFF) procedure while preserving the overall conformation of the model (electronic supplementary material, figure S4*b*) [40]. We had previously generated a molecular model of the caspase-like domain of *C. elegans* separase, which is globular in shape and extends over 50 Å in its longest dimension, and only fits into one subregion of the map, in the larger lobe [21]. We therefore annotated the two lobes as representing the N- and C-terminal regions of separase. Next, we fitted the death-like domain into a subregion of the C-terminal lobe adjacent to the caspase-like domain. We were unable to generate structural models for securin and the C-terminal region of separase (aa 1140–1262), and these sequences were not fitted into the map, which has regions of unmodelled density, particularly in the larger lobe. The caspase-like domain was modelled in complex with a substrate peptide based on *C. elegans* Scc1 (green, figure 5*b*) [21]. Although the active site of separase is exposed on the outer surface of the protein, access for large substrates is occluded by two neighbouring domains in the quaternary structure: a region of the N-terminal helical repeats (approx. aa 250–450), and an unassigned region of density that protrudes from the C-terminal lobe towards the N-terminal repeats (‘crooked tooth’).

## 3. Discussion

In this study, we report the production of recombinant separase–securin complex from *C. elegans* as well as its three-dimensional structure determined in negative stain from electron microscopic images.

We were not able to express separase alone or any truncation versions thereof in large scale using bacterial or insect cell expression systems. However, large amounts of soluble separase–securin complex were obtained in insect cell culture. A two-step purification protocol resulted in pure, monodisperse complex. This supports previous findings that the protease needs to be expressed with its inhibitor to accumulate and that securin functions as a chaperone for separase [11,13,14,17,19]. Further analysis of this complex using SEC–MALS revealed that the two components were present in equal stoichiometry.

### 3.1. A closed conformation of the separase–securin complex is consistent with its biochemical properties

Our analysis of the *C. elegans* complex is consistent with previous studies that showed, in homologues from diverse organisms, that the N-terminal region of separase and the C-terminal part of securin are important for complex formation [13,16,25,26]. It is also thought that the middle part of securin contributes significantly to the interaction, that securin also interacts with the C-terminal region of separase, and that the N- and C-terminal regions of separase interact [13,25,41]. These data suggest that the securin–separase complex must adopt a compact structure, in which interactions can occur between separase domains that are separated in primary sequence, with securin acting as a bridge between them. Electron microscopy studies at 21–24 Å resolution revealed an overall compact architecture of the *C. elegans* separase–securin complex. Homology models of individual separase domains could be docked into the three-dimensional structure in an arrangement that closely matches the protein density and is compatible with these proposed domain interactions.

The structure described here is the second three-dimensional structure of a separase–securin complex reported, and the first from an invertebrate source. The *C. elegans* separase–securin complex is of globular shape, with an intricate connectivity and a large central cavity that separates two domains. Strikingly, the presence of two domains is the only feature in common between the *C. elegans* model and the previously published *H. sapiens* model [26]. Although both density maps are nominally the same resolution, there are no obvious domain similarities in the two structural maps. The human complex shows an elongated shape, whereas the *C. elegans* model has a globular shape. Furthermore, there are clearly more features present in the *C. elegans* model, such as a number of protrusions similar in shape to helical repeats. This is particularly the case in the smaller lobe, which forms a lock washer motif that is closely compatible with the stacked HEAT repeat homology model predicted for residues 1–720 of separase. It is difficult to identify such features in the *H. sapiens* structure with the overall surface of the model being rather smooth and featureless. The models also have a very different overall appearance; the *C. elegans* structure is relatively globular with a large central cavity, whereas the *H. sapiens* structure is larger, elongated and without a central cavity.

Despite the low sequence identity in separase and securin homologues, it was unexpected to see such lack of structural conservation as both proteins belong to the same family of proteins and exert the same function. Cohesin and its component Scc1 are well conserved, and separase and securin are essential genes. Therefore, it could be expected that the evolutionary pressure on the complex to preserve its function was too high to give rise to this structural diversity. It might be the case that the two structures reflect genuine differences in the human and *C. elegans* complexes. However, it is tempting to speculate that the two structures might indicate two very different conformations for separase that could be associated with different functional states. Flexibility in the complex would not be surprising, because securin is an intrinsically disordered protein, and separase is a multi-domain protein composed mainly of helical repeats, which have well-documented flexibility [37,42–45].

Support for the existence of two biochemically distinct forms of the separase–securin complex is found in an elegant series of experiments using inhibitory peptides that mimic Scc1 substrate and covalently bind to the active site cysteine of separase [13,19]. These studies, using human and yeast proteins, showed that securin inhibits separase by blocking access of substrates to the active site, because pre-incubation of separase with securin prevented binding of peptide inhibitor. However, when inhibitor was bound to separase first, this did not markedly reduce the binding of securin to separase, indicating that securin can bind to separase by contacting residues outside the active site of separase, such as the N-terminal region. Based on these studies, it was proposed that securin does not directly compete with substrate for active site binding, but instead alters the conformation of separase, so that the active site is inaccessible to substrate. The ‘closed’ shape of the *C. elegans* complex bears a striking resemblance to the models proposed for securin inhibition of separase based on these biochemical data, in contrast to the ‘open’ shape of the human complex (figure 5*c*). We tentatively suggest the human complex might represent a partly active form of the complex that appeared during the multi-step purification. Indeed, the complex purified by Viadiu *et al.* [26] was partly active as evidenced by separase autocleavage. We hypothesize that securin holds the two lobes of separase together and that proteolysis of securin opens up the protein, allowing access to the active site. However, in this model, it is unclear how the interaction between the N-terminal repeats and the caspase-like domain enhances separase activity. An alternative model is that securin blocks the interaction between the two lobes and that, upon securin proteolysis, closure of the two lobes enables the N-terminal repeats to activate the caspase-like domain through an allosteric mechanism.

Further structural studies will be required to address many of the aspects of separase regulation that remain unresolved. For example, we still do not know why securin is required to stabilize separase, how securin inhibits separase or the precise details of protease activation. Addressing many of these questions will require an improvement in the resolution of the separase–securin structure by using cryo-EM or by X-ray crystallography, and through studies on separase in an active state. The difficulty in producing recombinant complex that is stable and amenable to structural studies has been a limiting factor in structural studies on this complex, and so the recombinant expression and purification of the *C. elegans* separase–securin complex will greatly facilitate future studies.

## 4. Material and methods

### 4.1. Expression plasmid construction

*Caenorhabditis elegans* separase was cloned into pET30-TEV for bacterial expression. For expression in Sf9 cells, all constructs were cloned into either pFBDM-His [46] or pFBDM-strepII, which was generated by inserting a double strepII tag containing a PreScission cleavage site into the PH promoter of pFBDM using *Bam*HI and *Bss*HII endonucleases. For co-expression of His_6_-tagged separase and MBP-tagged securin in insect cells, separase was placed under the p10 promoter of pFBDM-His using *Xma*I and *Kpn*I, whereas MBP-securin was amplified from pET30-MBP and inserted into the vector with *Bam*HI and *Not*I endonucleases.

### 4.2. Protein expression and purification

His_6_-tagged *C. elegans* separase was expressed in *E.* coli BL21 CodonPlus RIL (Stratagene) in LB media at 37°C overnight after induction with 0.3 mM IPTG. Cell pellets were stored at −80°C until required. Thawed cell pellet was resuspended in Tris buffer (50 mM Tris, 0.3 M NaCl, 1 mM MgCl_2_, 2 mM 2-mercaptoethanol, pH 7.5) containing 1 EDTA-free protease inhibitor cocktail tablet (Roche). Cells were lysed by sonication. Cleared lysates were applied to a 5 ml Ni–NTA superflow column (Qiagen), washed with Tris buffer containing 20 mM imidazole, and the bound proteins were eluted with a 0.02–0.5 M imidazole gradient. Pooled fractions containing His_6_-securin were cleaved using His_6_–TEV protease during overnight dialysis into TEV buffer (50 mM Tris, 0.1 M NaCl, 2.5 mM MgCl_2_, 2 mM 2-mercaptoethanol, pH 7.5). The cleaved protein was separated from His_6_–TEV using a Ni–NTA column, then further purified using SEC in Tris buffer.

Baculovirus production was carried out essentially as described in the Invitrogen guide to baculovirus expression vector systems [47]. Small-scale infections used 10 ml of Sf9 cells at a density of 1.2–1.8 × 10^6^ cells ml^−1^ were infected with P2 virus using multiplicity of infection of 2. The infected cells were cultured in Erlenmeyer flasks, using 10% of the nominal volume, at 27°C and 140 r.p.m., for 72 h. The cells were harvested by centrifugation, and the pellet stored at −80°C until needed or further processed immediately. The protocol was modified for large-scale infections: after infecting 400 ml of cell culture with P3 virus, the cells were cultured in roller bottles using 20% of the nominal volume.

Pelleted cells from small-scale cultures were sonicated on ice in PBS buffer containing 0.05% v/v nonidet and EDTA-free protease inhibitor cocktail set III (Calbiochem). Clarified cell lysate was mixed with approximately 25 µl of the appropriate affinity resin slurry (Ni superflow resin, Generon; amylose resin, NEB; StrepTactin superflow plus, Qiagen), incubated for 1 h at 4°C, and the resin was pelleted by centrifugation. The resin was washed four times in PBS buffer (containing 10 mM imidazole when working with His_6_-tagged proteins), and samples were analysed by SDS–PAGE and Western blot.

Pelleted cells from large-scale cultures were resuspended in either Tris buffer (50 mM Tris, 0.2–0.5 M NaCl, 2 mM β-mercaptoethanol, pH 7.0) or phosphate buffer (50 mM Tris, 0.5M NaCl, 2 mM β-mercaptoethanol, pH 7.0), supplemented with 0.05% v/v nonidet and protease inhibitors. Cells were lysed using a manual homogenizer, clarified and filtered. His_6_-tagged proteins and complexes were loaded onto a 5 ml Ni–NTA superflow column (Qiagen), washed with Tris or phosphate buffer containing 20 mM imidazole, and eluted with a 0.02–0.5 M imidazole gradient. StrepII-tagged proteins were loaded onto a 5 ml StrepTactin superflow plus column in phosphate buffer, and bound proteins were eluted with a phosphate buffer containing 2.5 mM d-desthiobiotin. MBP-securin/separase complexes were applied to amylose resin, washed with Tris or phosphate buffer and cleaved off the resin using TEV protease. The protein pool was concentrated and loaded onto a Superdex 200 16/60 SEC column or an analytical Superose 6 10/300 column (GE Healthcare) pre-equilibrated in Tris buffer, or phosphate buffer. Eluted fractions were analysed by SDS–PAGE and Western blots.

### 4.3. Circular dichroism spectroscopy

CD measurements of full-length His_6_-tagged *C. elegans* securin were carried out using a 0.75 mg ml^−1^ protein sample in a buffer comprising 35 mM Tris, 0.1 M NaCl, 1.25 mM MgCl_2_, 2.5 mM 2-mercaptoethanol, pH 7.5, which was diluted 10-fold in water, added to a 1 mm cuvette and kept at 25°C, whereas a 260 to 190 nm CD spectrum was recorded. The percentage distribution of α-helix, β-sheet and random coil in the data was calculated by using the CD data analysis programs CDSSTR and CONTINLL.

### 4.4. Thermal shift

5 µM protein sample in SEC buffer was diluted 10-fold in the same buffer containing imidazole. Next, SYBRO orange fluorescent dye was added to the samples in a final concentration of 1/1000 stock solution and the samples transferred to quartz cuvettes (Hellma). The samples were now heated from 4 to 80°C, in increments of 1°C at the rate of 2°C min^−1^, while simultaneously monitoring fluorescence changes with a Cary Eclipse fluorescence spectrophotometer (Agilent Technologies). The wavelengths for excitation and emission were 470 and 600 nm, respectively. To obtain the temperature midpoint for the protein unfolding transition, *T*_m_, the data were analysed using the mathematical software Prism. Here, a Boltzmann model was used to fit the fluorescence data to the following equation: *I* = (*A* + (*B* − *A*)/(1 + exp(*T*_m_ − *T*)/*C*), where *I* is the fluorescence intensity at temperature *T*, *A* and *B* are pretransitional and post-transitional fluorescence intensities, respectively, and *C* is the slope factor.

### 4.5. SEC–MALS

1 mg ml^−1^ protein samples were loaded onto a Superose 6 10/300 column pre-equilibrated in a buffer comprising 50 mM sodium phosphate, 100 mM NaCl, 20 mM NaF, 10% v/v glycerol, 5 mM 2-mercaptoethanol, 3 mM sodium azide, pH 8.0 at a flow rate of 0.5 ml min^−1^. The column was mounted on a Varian ProStar high-performance liquid chromatography controlled by the Galaxie software package. The scattered light intensity of the column eluent was recorded at 18 angles using a DAWN-HELEOS II laser light scattering detector (Wyatt Technology Corp., Santa Barbara, CA). The refractive index change was detected using an OPTILAB-rEX differential refractometer (Wyatt Technology Corp.). The wavelength of the laser in the DAWN-HELEOS II, and the light source in the OPTILAB-rEX was 658 nm. The weight-averaged molecular mass of protein contained in chromatographic peaks was determined using the ASTRA software version 5 (Wyatt Technology Corp.).

### 4.6. Electron microscopic and image analysis

Purified separase/securin complex was loaded on to carbon-coated glow-discharged quantifoil grids and negatively stained with 2% w/v uranyl acetate. Micrographs were collected on a Tecnai F20 electron microscope operating at 200 kV and recorded using a 4 k × 4 k pixel CCD camera (Tietz) at a nominal 62 000 × magnification resulting in a pixel size of 2.914 Å at specimen level. The focal level was chosen such that the first minimum of the contrast transfer function was placed at 18 Å. Low dose settings were used but with an electric dose of approximately 100 *e-*/Å2.

Single molecular views were chosen manually from micrographs using the graphical program Boxer, which is a part of the EMAN software [48]. Data stacks were high-pass filtered to 250 Å, masked with a circle (radius 0.9), and normalized to zero mean and standard deviation of 2 in IMAGIC-5 [49]. The data were subsequently low-pass filtered to 15 Å with the ‘fq’ command in SPIDER, choosing the Fermi filter option [50].

Initial classification was carried out using an automated reference-free EMAN procedure (refine2d). Here, standard parameters used, for datasets of about 1800–6000 particles, were 300 initial class averages, seven iterations and 1000 final class averages. The class averages were prepared for further processing by applying a soft mask using the ‘mask-im’ command in IMAGIC. A preliminary three-dimensional model was constructed in IMAGIC and refined through iterative cycles of reprojection, projection matching, adjustment of the number of classes, and visual inspection of the agreement between class averages and the corresponding reprojections.

## Supplementary Material

Biochemical analysis of the separase-securin complex. Characterisation of the refined separase-securin model. Estimation of the resolution of the refined separase-securin model. Modelling of the N-terminal domain of separase.

## Data accessibility

The electron density map was deposited in the EMDataBank with accession code EMD-3384.
