# Supplementary material for: A closed conformation of the Caenorhabditis elegans separase–securin complex
Source: Open Biol. 2016 Apr 13;6(4):160032. doi: 10.1098/rsob.160032 (PMC4852461; doi:10.1098/rsob.160032)
Supplement: Biochemical analysis of the separase-securin complex. Characterisation of the refined separase-securin model. Estimation of the resolution of the refined separase-securin model. Modelling of the N-terminal domain of separase. [file rsob160032supp1.pdf]

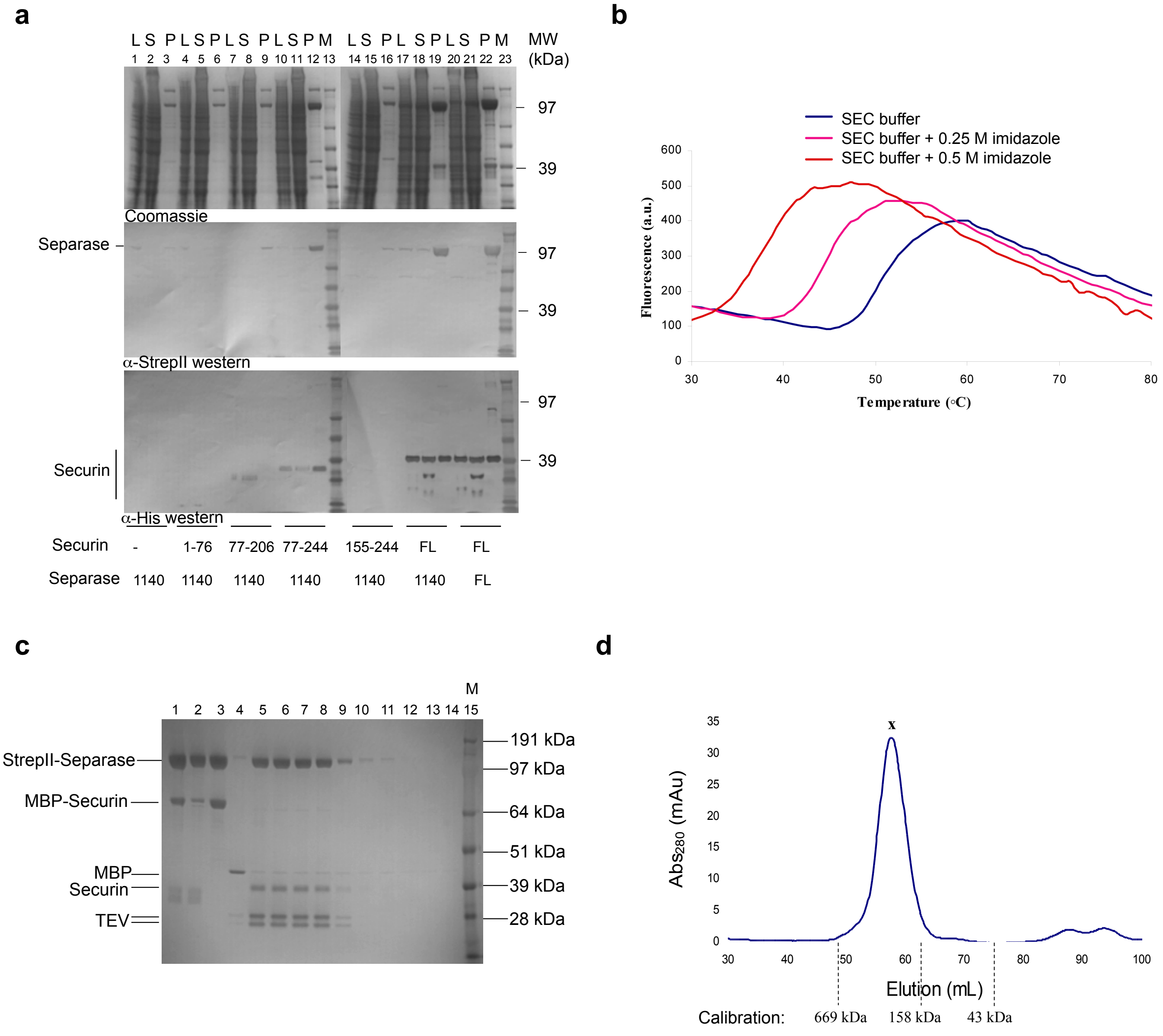

Figure S1: Biochemical analysis of the separase-securin complex. a) Full data used to generate figure 2b. b) Denaturation curves from a thermal shift assay using SYPRO Orange to monitor the unfolding of the separase-securin complex. SEC buffer was 50mM sodium phosphate buffer, 0.1M NaCl, 20mM NaF, 5mM β-mercaptoethanol, pH 8.0. c) Second purification step: SDS-PAGE analysis of the cleavage of the bound MBP-tagged securin from the amylose resin using TEV protease. The separase-securin complex (lane 1) was incubated with amylose beads, resulting in unbound (lane 2) and bound (lane 3) fractions. After cleavage and washing, MBP is still bound to the beads as indicated by a band at around 42kDa (lane 4). Lanes 5 to 14 show fractions of soluble StrepII-separase and securin after cleavage. d) Third purification step: elution profile of StrepII-separase and securin from SEC. The elution profile shows one peak indicating a monodisperse solution of separase-securin complex. Masses obtained from calibration runs are shown for comparison.

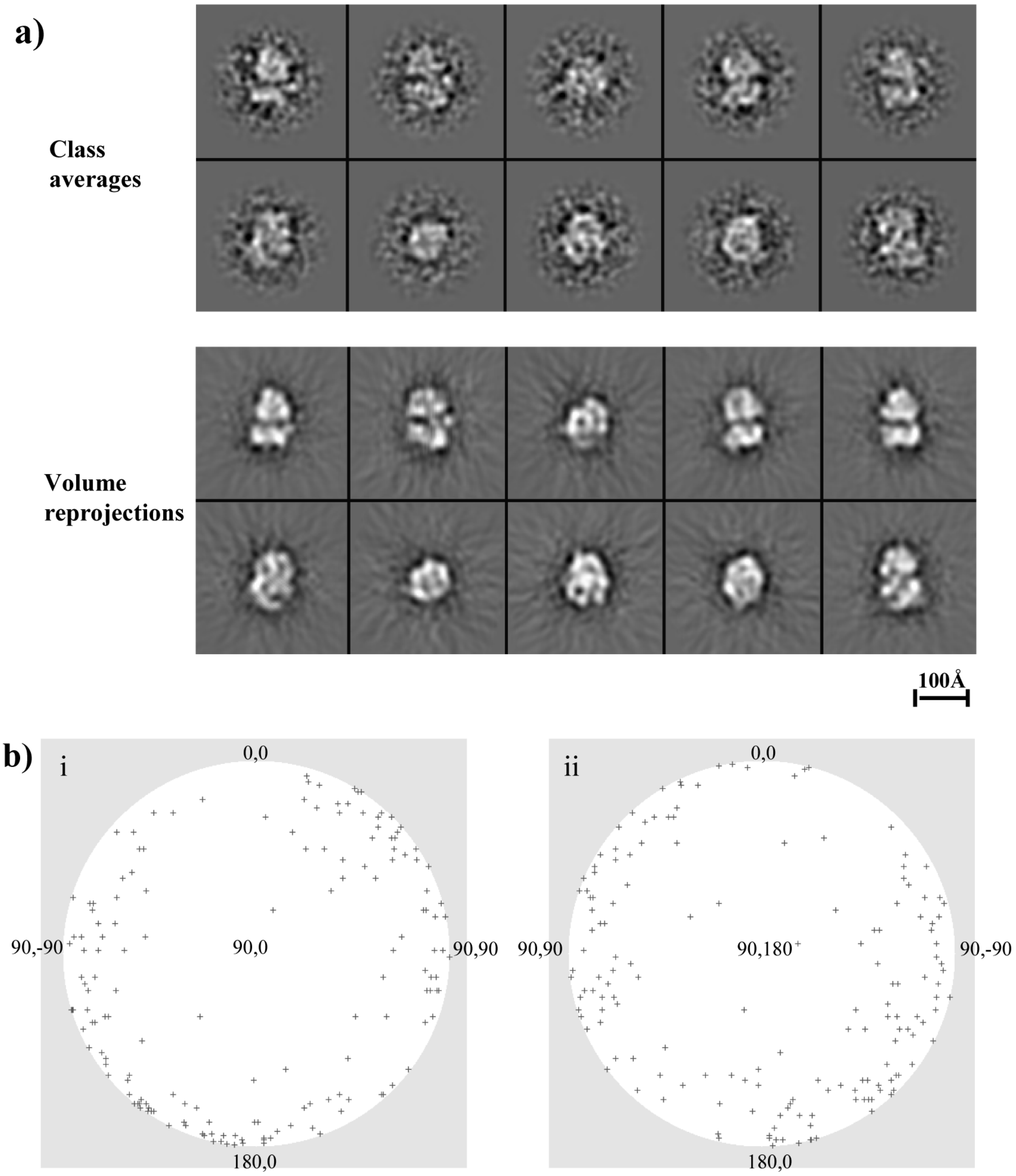

Figure S2: Characterisation of the refined separase-securin model
a) Representative selection of refined class averages (top) and corresponding reprojections of the 3D structure (bottom). b) An angular distribution plot of the class averages of the refined separase/securin model demonstrates the almost random orientations of the separase-securin complex on the carbon surface. i, front hemisphere. ii, back hemisphere. The first angle is the angle between the North pole and the latitude line of the determined orientation for a particular view. The second angle defines the longitude of the orientation. The different orientations of the class averages are represented by crosses.

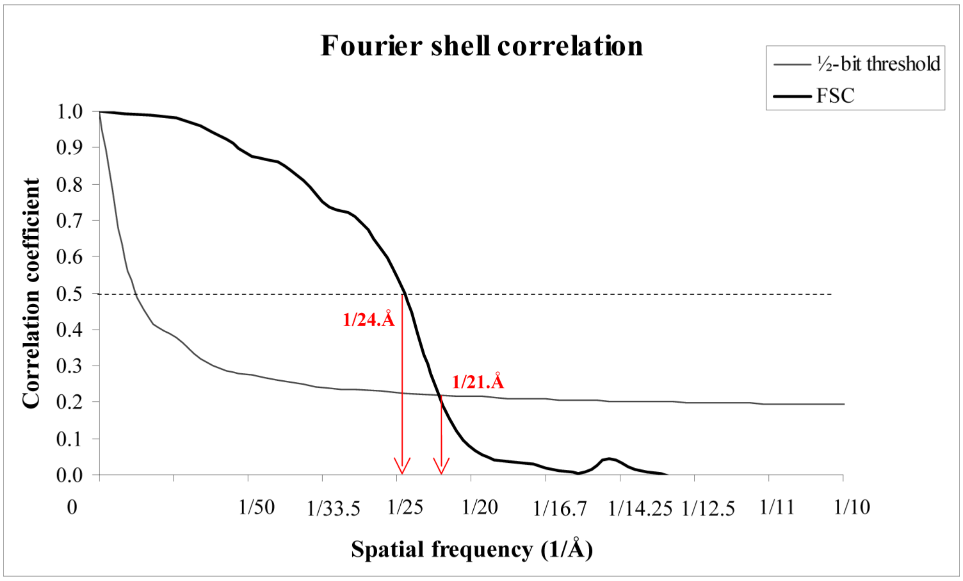

Figure S3: Estimation of the resolution of the refined separase-securin model
Fourier shell correlation based resolution estimates of the separase-securin reconstruction from IMAGIC indicate 24Å based on the 0.5 threshold and 21Å based on the ½-bit threshold (red arrows).

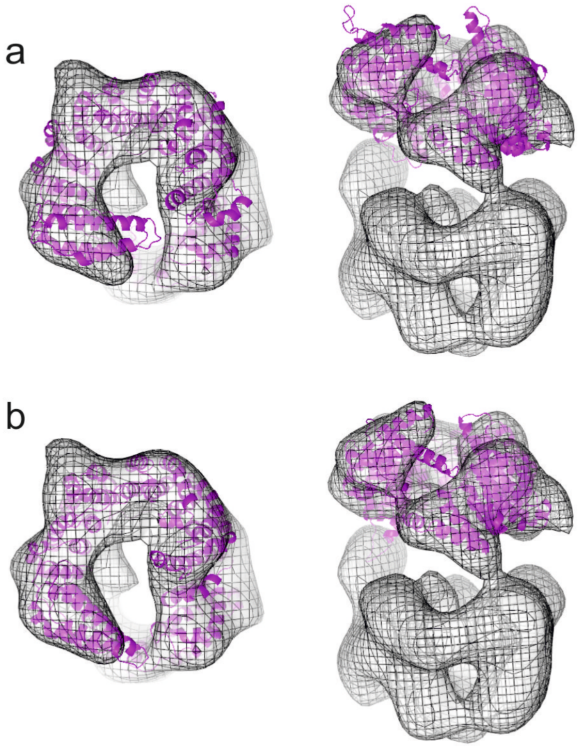

Figure S4: Modelling of the N-terminal domain of separase. a) A homology model of the N-terminal domain of separase (residues 1-720) generated by I-TASSER was found to correspond quite closely to the small lobe of the 3D map of the separase-securin complex, matching the lock-washer configuration. b) Flexible fitting of the model with MDFF improved the agreement with the density while retaining the general features the model.
